# Interleukin-6 Receptor Gene rs1800795 Polymorphism and Expression of Interleukin-6 in Gingival Tissue in Patients with Periodontitis

**DOI:** 10.3390/microorganisms12101954

**Published:** 2024-09-27

**Authors:** Małgorzata Mazurek-Mochol, Tobias Bonsmann, Damian Malinowski, Karol Serwin, Michał Czerewaty, Krzysztof Safranow, Andrzej Pawlik

**Affiliations:** 1 Department of Periodontology, Pomeranian Medical University in Szczecin, 70-111 Szczecin, Poland; malgorzata.mazurek@poczta.onet.pl (M.M.-M.); tobias.bonsmann1@gmail.com (T.B.); 2 Department of Pharmacokinetics and Therapeutic Drug Monitoring, Pomeranian Medical University in Szczecin, 70-111 Szczecin, Poland; damian.malinowski@pum.edu.pl; 3 Department of Infectious, Tropical Diseases and Immune Deficiency, Pomeranian Medical University in Szczecin, 70-111 Szczecin, Poland; karol.serwin@pum.edu.pl; 4 Department of Physiology, Pomeranian Medical University in Szczecin, 70-111 Szczecin, Poland; michal.czerewaty@wp.pl; 5 Department of Biochemistry and Medical Chemistry, Pomeranian Medical University, 70-111 Szczecin, Poland; chrissaf@mp.pl

**Keywords:** periodontitis, interleukin-6 receptor, polymorphism, expression, gingival tissue

## Abstract

Periodontitis is a multifactorial inflammatory disease. This chronic periodontal disease is caused by a bacterial infection in the gums, which triggers a host inflammatory response. To eliminate the bacterial infection, immune response mechanisms are activated, leading to inflammation and damage to the periodontal tissues. This process involves many cytokines, including IL-6, a cytokine with antibacterial properties. An ongoing bacterial infection in the periodontal tissues leads to its excessive production, which increases inflammation. In this study, we examined *IL-6* receptor gene rs1800795 polymorphism in patients with periodontitis in comparison with healthy subjects, as well as the correlation between rs1800795 genotypes and clinical parameters. Additionally we examined the expression of *IL-6* in gingival tissue in patients with periodontitis and control subjects, as well as the correlation between gingival expression of *IL-6* and clinical parameters. This study included 200 patients with periodontitis and 158 healthy subjects as the control group. Biopsy specimens of gingival tissue in which *IL-6* expression was detected were taken from 14 patients with periodontitis and 8 controls who had undergone minor surgery. There were no statistically significant differences in the distribution of *IL-6* rs1800795 genotypes and alleles between patients with periodontitis and control subjects. There were also no statistically significant correlations between *IL-6* rs1800795 genotypes and clinical parameters in patients with periodontitis. There were no differences in *IL-6* expression in the gingival tissue between patients with periodontitis and controls. There was also no correlation between *IL-6* expression in the gingival tissue of patients with periodontitis and clinical parameters. In the control group, *IL-6* expression in gingival tissue correlated negatively with the approximal plaque index, which reflects the size of bacterial plaques. The results of our study suggest a protective role for IL-6 against bacterial growth in the periodontal tissue. However, it should be noted that several parameters directly or indirectly affect the accumulation of bacterial plaque.

## 1. Introduction

Periodontitis is a multifactorial inflammatory disease. This chronic periodontal disease is caused by a bacterial infection within the gums, which triggers a host inflammatory response. To eliminate the bacterial infection, immune response mechanisms are activated, leading to inflammation and damage to the periodontal tissues. An ongoing bacterial infection in the periodontal tissues leads to excessive production of proinflammatory cytokines, which increases inflammation. If not recognized and treated at an early stage, it irreversibly damages the periodontium, including loss of periodontal ligaments and alveolar bone, ultimately leading to the loss of single or multiple teeth [1]. In addition to the pathogenic and destructive processes that affect the periodontium, periodontitis is well known for its close correlation with the health status of patients suffering from other systemic diseases, such as diabetes [2], cardiovascular disease [3,4,5], cancer [6], and chronic respiratory diseases [7]. While the basic features of periodontitis include clinical attachment loss (CAL) and radiographically assessable alveolar bone loss [8], the diagnosis is now broader and includes not only a variety of clinical but also laboratory investigations. For decades, the classification of periodontal disease included a division into localized and generalized periodontitis. Local and systemic risk factors, such as other systemic diseases and/or habits such as smoking, have not been mapped and accounted for in different degrees of severity and progression. The 2017 World Workshop on Classification of Periodontal and Peri-implant Diseases set out to design a case definition system that could be implemented in clinical practice, research, and epidemiological surveillance. With this new 2017 system, each patient can be defined by the specific complexity of the disease, and progression or improvement after treatment is easier to determine. While numerous clinical indicators such as bleeding on examination (BoP), clinical attachment level (CAL), probing pocket depth (PPD), and many others have been firmly integrated into the assessment of the severity and progression of periodontitis for years, laboratory tests of gingival crevice fluid and blood samples have recently gained importance. Several proinflammatory cytokines are involved in the pathogenesis of periodontitis, causing the development of inflammation.

One of these interleukins that plays a role in the pathophysiology of periodontitis is interleukin 6 (IL-6). Previous studies indicate the importance of IL-6 in initiating both local and general inflammatory processes [9,10]. Previous studies have shown increased IL-6 expression in saliva and gingival fluid in patients with periodontitis. IL-6 acts through its receptors [11,12]. A polymorphism has been found in the gene encoding the receptor for IL-6, which may alter its expression.

In this study, we examined *IL-6* receptor gene rs1800795 polymorphism in patients with periodontitis in comparison with healthy subjects, as well as the correlation between rs1800795 genotypes and clinical parameters. Additionally we examined the expression of *IL-6* in gingival tissue in patients with periodontitis and control subjects, as well as the correlation between gingival expression of *IL-6* and clinical parameters.

## 2. Materials and Methods

### 2.1. Patients

This study included 200 Caucasian patients with periodontitis (84 male, 116 female, 130 nonsmokers, 70 smokers, age 49.9 ± 8.7 years, periodontal probing depth 4.4 ± 2.3 mm, approximal plaque index 73.0 ± 21.0%, clinical attachment level 5.0 ± 2.4 mm, bleeding on probing 57.7 ± 25.5%, number of teeth present 20.8 ± 6.5) diagnosed according to the 2017 World Workshop on Classification of Periodontal and Peri-implant Diseases guidelines and 158 healthy subjects as the control group (56 male 102 female, 124 nonsmokers, 34 smokers, age 45.4 ± 10.2 years periodontal probing depth 1.6 ± 0.5 mm, approximal plaque index 35.7 ± 20.1%, clinical attachment level 0.4 ± 1.2 mm, bleeding on probing 6.6 ± 11.4%, number of teeth present 29.2 ± 4.2). Exclusion criteria included systemic and autoimmune diseases, diabetes, and cancers, as well as treatment with antimicrobial, anti-inflammatory, and immunosuppressive drugs.

Biopsy specimens of gingival tissue from vestibular area in which *IL-6* expression was detected were taken from 14 patients with periodontitis and 8 controls who had undergone minor surgery.

This study was approved by the local ethics committee (KB-0012/134/18) and conducted per the Declaration of Helsinki. Patients were informed about the study, and their written consent was obtained.

### 2.2. Periodontal Examination

Conventional clinical indices of periodontal health status, including bleeding on probing (BoP), probing pocket depth (PPD), the approximal plaque index (API), and the clinical attachment level (CAL), were used for periodontal evaluation. All measurements were performed under consistent conditions in the same dental clinic. A UNC-15 Color-Coded Probe was used with circa 0.20 N of pressure for all explorations. To assess probing pocket depth (PPD) and clinical attachment level (CAL), which were probed on 6 sides per tooth, a UNC 15 periodontal probe with a 1 mm calibration (HU-Friedy Mfg. Co., Inc., Chicago, IL, USA) was used.

### 2.3. Genotyping

Genomic DNA was extracted from whole blood collected in EDTA tubes using the GeneMATRIX Quick Blood DNA Purification Kit (EURx, Gdansk, Poland). Identification of all examined polymorphisms in the *IL-6* gene was performed using the TaqMan SNP genotyping assay (Applied Biosystems, Waltham, MA, USA). The reaction was performed in duplicate on a 7500 Fast Real-Time PCR Detection System (Applied Biosystems, Waltham, MA, USA).

### 2.4. Real-Time PCR

Total mRNA was extracted from gingival tissue samples (50–100 mg) from vestibular area using the RNeasy Mini Kit (Qiagen, Hilden, Germany). The RNA (0.5 µg) was reverse-transcribed with the RevertAid First Strand cDNA synthesis kit (Thermo Scientific, Waltham, MA, USA) according to the manufacturer’s protocol. Quantitative expression analysis of *IL-6* was performed using qRT-PCR on an ABI PRISM^®^ Fast 7500 Sequence Detection System (Applied Biosystems, Waltham, MA, USA). The thermal cycling conditions were as follows: 95 °C for 15 s, followed by 40 cycles at 95 °C for 15 s and 60 °C for 1 min. Expression levels of *IL-6* and endogenous control were determined by CT values measured in duplicate. Data normalization was performed based on the reference gene β2-microglobulin. To calculate the values, the 2^−ΔCt^ method was used. The sequences of used primers were prepared according to the sequence information obtained from the NCBI database and were synthesized by Oligo.pl (IBB PAN, Warszawa, Poland) manufacturer.

### 2.5. Statistical Analysis

The consistency of the genotype distribution with Hardy–Weinberg equilibrium (HWE) was assessed with Fisher’s exact test. Chi-square and Fisher’s exact tests were used to compare genotype and allele distributions between study groups.

Distributions of quantitative variables differed significantly from a normal distribution (Shapiro–Wilk test); thus, nonparametric tests were used. Values were compared between genotype groups with the Kruskal–Wallis or Mann–Whitney test, and correlations within groups were assessed with the Spearman rank correlation coefficient. A result was considered statistically significant at *p* < 0.05.

## 3. Results

### 3.1. IL-6 rs1800795 Polymorphism

The distribution of *IL-6* rs1800795 genotypes was in Hardy–Weinberg equilibrium.

There were no statistically significant differences in the distribution of *IL-6* rs1800795 genotypes and alleles between patients with periodontitis and control subjects (Table 1). There were also no statistically significant differences in the distribution of studied genotypes and alleles between smoking patients and controls as well as nonsmoking patients and control subjects (Table 2 and Table 3).

There were also no statistically significant associations between IL-6 rs1800795 genotypes and clinical parameters in patients with periodontitis (Table 4).

### 3.2. IL-6 Expression in Gingival Tissue

**Figure 1:**
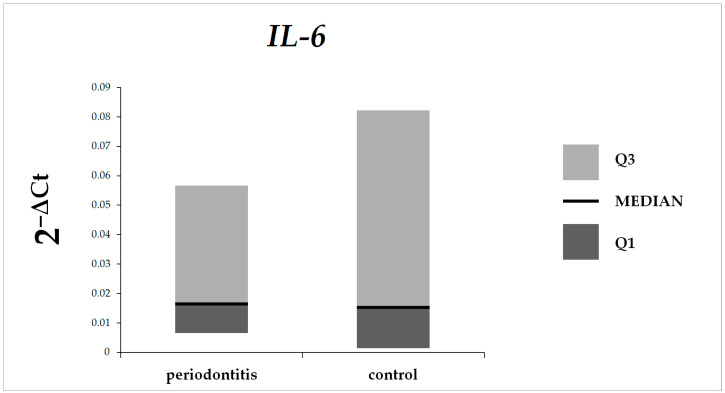
*IL-6* expression in gingival tissue in patients with periodontitis and control group.

There were no statistically significant differences in *IL-6* expression in gingival tissue between patients with periodontitis and control subjects (Figure 1).

There were also no statistically significant correlations between *IL-6* expression in gingival tissue and clinical parameters in patients with periodontitis (Table 5).

In healthy subjects, *IL-6* expression in gingival tissue correlated significantly with API values. There was no statistically significant correlation between *IL-6* expression in gingival tissue and other clinical parameters (Table 6).

## 4. Discussion

This study aimed to evaluate the association of the *IL-6* receptor gene rs1800795 polymorphism with the risk of periodontitis and selected clinical parameters, as well as to assess the expression of *IL-6* in gingival tissue in patients with periodontitis. The polymorphism studied was not associated with the increased the risk of periodontitis, nor was its correlation with clinical parameters. Also there were no differences in *IL-6* expression in the gingival tissue between patients with periodontitis and controls. There was also no correlation between *IL-6* expression in the gingival tissue of patients with periodontitis with clinical parameters. In the control group, *IL-6* expression in gingival tissue correlated negatively with the approximal plaque index. This may indicate a protective role for IL-6 against bacterial growth in the periodontal tissue.

Previous studies have demonstrated an important role for IL-6 in the development of periodontitis, showing elevated levels of this cytokine in the saliva and gingival fluid of patients with periodontitis [12,13,14,15,16,17,18]. IL-6 is a proinflammatory cytokine that plays a multifaceted role both in protection against bacterial infections and in the development of inflammatory and autoimmune diseases [10,19]. Periodontitis is a process initiated by a bacterial infection in the periodontal tissues, which results in the induction of immune processes and inflammation [20,21,22]. Numerous proinflammatory cytokines are involved in this process, including IL-6. Different inflammatory response cells play a role in the development of the inflammatory process within the periodontal tissues, infiltrating the periodontal tissues and acting as a source of numerous mediators that increase the development of inflammation and cause the destruction of the periodontal tissues [23]. Previous studies have shown the highest concentrations of proinflammatory mediators in the gingival fluid and saliva of patients with periodontitis [24]. In our study, there were no significant differences in *IL-6* expression in gingival tissue between patients with periodontitis and controls. IL-6 is mainly produced by immune cells infiltrating periodontal tissues and is secreted into the gingival fluid [11].

In the group of patients with periodontitis, we did not demonstrate an association of gingival *IL-6* expression with clinical parameters. This may reflect the complex pathogenesis of the inflammatory process in periodontitis, which is influenced by several cytokines and chemokines, such as IL-1, TNF-α, and IL-17, and interactions between them. In periodontitis, various mediators are interrelated and interact to increase inflammation and cause tissue destruction [25,26]. It therefore appears that the effect of a single cytokine on clinical parameters is small. In contrast, we found a negative correlation between gingival *IL-6* expression and the approximal plaque index, which shows the size of the plaque and, thus, the intensity of bacterial colonization in the periodontal area [27]. IL-6 is a cytokine with multidirectional effects, including antimicrobial activity [10].

Previous studies have shown an association of *IL-6* gene polymorphisms with the development of periodontitis [28,29,30,31,32]. We did not demonstrate an association between *IL-6* receptor gene rs1800795 polymorphisms and the risk of periodontitis and a correlation of this polymorphism with selected clinical parameters in patients with periodontitis. This may be due to the complexity of periodontitis pathogenesis and the involvement of multiple environmental and genetic factors, where the impact of a single polymorphism is small. The *IL-6* rs1800795 polymorphism may have different effects in different pathologies such as diabetes mellitus and may also vary depending on the ethnic origins of patients, such as Asians and mixed populations [33].

In conclusion, the results of our study did not demonstrate a statistically significant contribution of the *IL-6* receptor gene polymorphism to the pathogenesis of periodontitis. We also did not demonstrate statistically significant differences in *IL-6* expression in the gingival tissue of patients with periodontitis and controls, as well as correlations of *IL-6* with clinical parameters in patients with periodontitis. We only found a negative correlation of *IL-6* expression in gingival tissue with the approximal plaque index in healthy subjects, which may indicate a protective role for IL-6 against the development of bacterial colonization and plaque.

The lack of demonstration of differences in *IL-6* expression in gingival tissue between patients with periodontitis and healthy controls does not negate the important role of this cytokine in the development of periodontitis but only seems to confirm previous observations that IL-6 secreted by immune cells into the gingival fluid plays an essential role in the development of periodontitis.

It should be noted that IL-6 is a component of a complex cascade of many proinflammatory cytokines involved in the development of periodontitis. The expression of cytokines is not static, but can change with an increase and then a decrease in the dysbiosis of the microbiome, depending on the severity of virulence of pathogens and the degree of their diversity.

An important limitation of our study is the lack of explanation of regulatory interactions between the gingival mucosa and the oral microbiome, as well as the lack of analysis of factors affecting *IL-6* and its receptor expression, such as signaling pathways inducing its transcriptional activity. It is also important to note the possible influence of the microbiome on *IL-6* and its receptor expression.

## 5. Conclusions

The results of our study indicate a protective role for IL-6 against bacterial growth in the periodontal tissue. IL-6 is considered a proinflammatory cytokine that facilitates the transition from acute to chronic inflammation. As a result, this transition promotes the progression of periodontitis. This also explains the effect of IL-6 on bone metabolism, as IL-6 is able to induce osteoclast formation.

## Figures and Tables

**Table 1:** The distribution of *IL-6* rs1800795 genotypes in periodontitis patients (PD patients) and control group.

	PD Patients (n = 200)		Control Group (n = 158)		*p* ^a^		*p* ^b^	OR (95% CI)
	n	%	n	%				
*IL-6 rs1800795*								
genotype								
GG	48	24.00%	46	29.12%	0.35	CC + GC vs. GG	0.28	1.30 (0.81–2.09)
GC	100	50.00%	80	50.63%		CC vs. GC + GG	0.21	1.38 (0.84–2.28)
CC	52	26.00%	32	20.25%		CC vs. GG	0.17	1.56 (0.86–2.83)
						GC vs. GG	0.52	1.20 (0.73–1.98)
						CC vs. GC	0.35	1.30 (0.77–2.21)
*IL-6 rs1800795*								
allele								
G	196	49.00%	172	54.43%				
C	204	51.00%	144	45.57%		C vs. G	0.15	1.24 (0.93–1.67)

^a^ χ^2^ test. ^b^ Fisher exact test. HWE: examined group *p* = 1.00, control group *p* = 0.80.

**Table 2:** The distribution of *IL-6* rs1800795 genotypes in periodontitis patients (PD patients) and controls in nonsmokers group.

	PD Patients (n = 130)		Control Group (n = 124)		*p* ^a^		*p* ^b^	OR (95% CI)
	(Nonsmokers)		(Nonsmokers)					
	n	%	n	%				
*IL-6 rs1800795*								
genotype								
GG	34	26.15%	37	29.84%	0.50	CC + GC vs. GG	0.58	1.20 (0.69–2.08)
GC	63	48.46%	63	50.81%		CC vs. GC + GG	0.29	1.42 (0.78–2.57)
CC	33	25.39%	24	19.35%		CC vs. GG	0.29	1.50 (0.74–3.02)
						GC vs. GG	0.88	1.09 (0.61–1.95)
						CC vs. GC	0.34	1.38 (0.73–2.59)
*IL-6 rs1800795*								
allele								
G	131	50.38%	137	55.24%				
C	129	49.62%	111	44.76%		C vs. G	0.29	1.22 (0.86–1.72)

^a^ χ^2^ test. ^b^ Fisher exact test.

**Table 3:** The distribution of *IL-6* rs1800795 genotypes in periodontitis patients (PD patients) and controls in smokers group.

	PD Patients (n = 70)		Control Group (n = 34)		*p* ^a^		*p* ^b^	OR (95% CI)
	(Smokers)		(Smokers)					
	n	%	n	%				
*IL-6 rs1800795*								
genotype								
GG	14	20.00%	9	26.47%	0.75	CC + GC vs. GG	0.46	1.44 (0.55–3.77)
GC	37	52.86%	17	50.00%		CC vs. GC + GG	0.81	1.21 (0.47–3.14)
CC	19	27.14%	8	23.53%		CC vs. GG	0.56	1.53 (0.47–4.95)
						GC vs. GG	0.60	1.40 (0.51–3.86)
						CC vs. GC	1.00	1.09 (0.40–2.98)
*IL-6 rs1800795*								
allele								
G	65	46.43%	35	51.47%				
C	75	53.57%	33	48.53%		C vs. G	0.56	1.22 (0.69–2.19)

^a^ χ^2^ test. ^b^ Fisher exact test.

**Table 4:** The associations between selected clinical parameters (mean values ± SD) and *IL-6* rs1800795 genotypes.

	GG	GC	CC	GG vs. GC * p-Value	GG vs. CC * p-Value	CC vs. GC * p-Value	GG vs. GC + CC * p-Value	CC vs. GC + GG * p-Value
API (%)	72.19 ± 18.99	74.63 ± 20.97	72.67 ± 19.33	0.385	0.860	0.465	0.496	0.652
BoP (%)	61.35 ± 24.59	57.10 ± 23.82	56.50 ± 27.98	0.313	0.396	0.724	0.290	0.550
PPD	4.67 ± 1.09	4.60 ± 1.29	4.42 ± 1.09	0.576	0.341	0.581	0.431	0.435
CAL	5.22 ± 1.60	5.06 ± 1.58	4.92 ± 1.43	0.677	0.309	0.523	0.475	0.384

API—approximal plaque index, BoP—bleeding on probing, PPD—periodontal probing depth, CAL—clinical attachment loss. * Mann–Whitney *U*-test.

**Table 5:** Correlation between gingival IL-6 expression and selected clinical parameters in patients with periodontitis.

Parameter	R	*p*
API	0.17822	0.542
BoP	−0.44884	0.107
PPD	−0.17802	0.542
CAL	0.17822	0.542

R—correlation coefficient, *p*—probability value, PPD—periodontal probing depth, CAL—clinical attachment level, API—approximal plaque index, BoP—bleeding on probing.

**Table 6:** Correlation between gingival IL-6 expression and selected clinical parameters in the control group.

Parameter	R	*p*
API	−0.72294	0.042
BoP	−0.63434	0.091
PPD	−0.64286	0.085
CAL	−0.17964	0.670

R—correlation coefficient, *p*—probability value, PPD—periodontal probing depth, CAL—clinical attachment level, API—approximal plaque index, BoP—bleeding on probing.

## Data Availability

The original contributions presented in the study are included in the article, further inquiries can be directed to the corresponding author.
